# Characterization of *Salmonella* Type III Secretion Hyper-Activity Which Results in Biofilm-Like Cell Aggregation

**DOI:** 10.1371/journal.pone.0033080

**Published:** 2012-03-08

**Authors:** Matthew E. Jennings, Laura N. Quick, Nicha Ubol, Sally Shrom, Norman Dollahon, James W. Wilson

**Affiliations:** 1 Department of Biology, Villanova University, Villanova, Pennsylvania, United States of America; 2 Department of Biological Sciences, University of Arkansas, Fayetteville, Arkansas, United States of America; 3 Department of Pathology and Laboratory Medicine, Children's Hospital of Philadelphia, Philadelphia, Pennsylvania, United States of America; University of Osnabrueck, Germany

## Abstract

We have previously reported the cloning of the *Salmonella enterica* serovar Typhimurium SPI-1 secretion system and the use of this clone to functionally complement a ΔSPI-1 strain for type III secretion activity. In the current study, we discovered that *S.* Typhimurium cultures containing cloned SPI-1 display an adherent biofilm and cell clumps in the media. This phenotype was associated with hyper-expression of SPI-1 type III secretion functions. The biofilm and cell clumps were associated with copious amounts of secreted SPI-1 protein substrates SipA, SipB, SipC, SopB, SopE, and SptP. We used a C-terminally FLAG-tagged SipA protein to further demonstrate SPI-1 substrate association with the cell aggregates using fluorescence microscopy and immunogold electron microscopy. Different *S.* Typhimurium backgrounds and both flagellated and nonflagellated strains displayed the biofilm phenotype. Mutations in genes essential for known bacterial biofilm pathways (*bcsA*, *csgBA*, *bapA*) did not affect the biofilms formed here indicating that this phenomenon is independent of established biofilm mechanisms. The SPI-1-mediated biofilm was able to massively recruit heterologous non-biofilm forming bacteria into the adherent cell community. The results indicate a bacterial aggregation phenotype mediated by elevated SPI-1 type III secretion activity with applications for engineered biofilm formation, protein purification strategies, and antigen display.

## Introduction

Type III secretion systems (T3SS) are found in many Gram negative bacteria including the genera *Salmonella*, *Shigella*, *Escherichia*, *Yersinia*, *Burkholderia*, *Pseudomonas*, *Chlamydia*, *Vibrio*, *Erwinia* and *Xanthomonas* [1], [2]. These systems are capable of secretion of substrate proteins into the extracellular milleu and translocation of effector proteins to the inside of eukaryotic cells via a syringe-like needle apparatus that extends from the bacterial surface [1], [2]. The genes encoding T3SS express several families of proteins that associate in the Gram negative envelope to form a macromolecular machine composed of inner and outer rings, a connecting rod, the needle structure, a complex of inner membrane-spanning proteins, and cytoplasmically-associated proteins which include an ATPase [1], [2], [3]. Translocation proteins responsible for forming a conduit that allows substrate transit across eukaryotic membranes are secreted by T3SS and can display effector activity inside eukaryotic host cells [4], [5], [6]. Dedicated effector proteins that target host cell functional pathways and drive host cell alterations are also substrates for T3SS [7], [8], [9]. The above protein “parts” of the T3SS act in concert to facilitate intimate interactions between bacterial and eukaryotic cells that allow cell invasion, phagosome remodeling, intracellular survival, host cell surface alterations, and manipulation of signaling pathways [1], [2], [7], [8], [9], [10], [11], [12].

*Salmonella* pathogenicity island 1 (SPI-1) is a horizontally-acquired set of approximately 45 genes that encodes a T3SS in *Salmonella enterica* serovars [11], [13]. The main function of the SPI-1 T3SS appears to be the alteration of host cell signaling pathways to allow invasion of *S. enterica* into eukaryotic host cells [11], [12]. The SPI-1 T3SS secretes proteins SipB, SipC, and SipD that facilitate translocation of effector substrates into eukaryotic cells where pathways related to actin rearrangement, cell death, and other functions are altered by direct interaction of effectors with key eukaryotic signaling proteins [4], [6], [7], [11], [12], [14]. The effector substrates include SipA, SipB, SipC, SptP, and AvrA (encoded within SPI-1) and SopE, SopE2, and SopB (encoded outside SPI-1) [4], [6], [7], [11]. To study the SPI-1 T3SS outside the context of the *S. enterica* chromosome, we cloned the entire system from *S. enterica* serovar Typhimurium onto the plasmid vector R995 [15]. This clone is able to complement a ΔSPI-1 *S.* Typhimurium strain for substrate secretion and eukaryotic cell invasion [15]. In the present study, we discovered that *S.* Typhimurium cultures containing cloned SPI-1 display an adherent biofilm and cell clumps in the media. This report describes the characterization of this phenotype and reveals a bacterial aggregation activity mediated by elevated SPI-1 type III secretion that occurs independently of eukaryotic cells. Interestingly, these observations are also consistent with a previously reported pellicle phenotype associated with the *Erwinia chrysanthemi* T3SS [16]. Overall, this activity has applications for engineered biofilm formation, protein purification strategies, antigen display, and interactions with eukaryotic host cells. In addition, the results indicate the potential for elevated T3SS activity to be a productive line of research that may yield novel previously unexplored uses for these systems.

## Results

### Strains containing R995 + SPI-1 form biofilm-like cell aggregates

**Figure 1:**
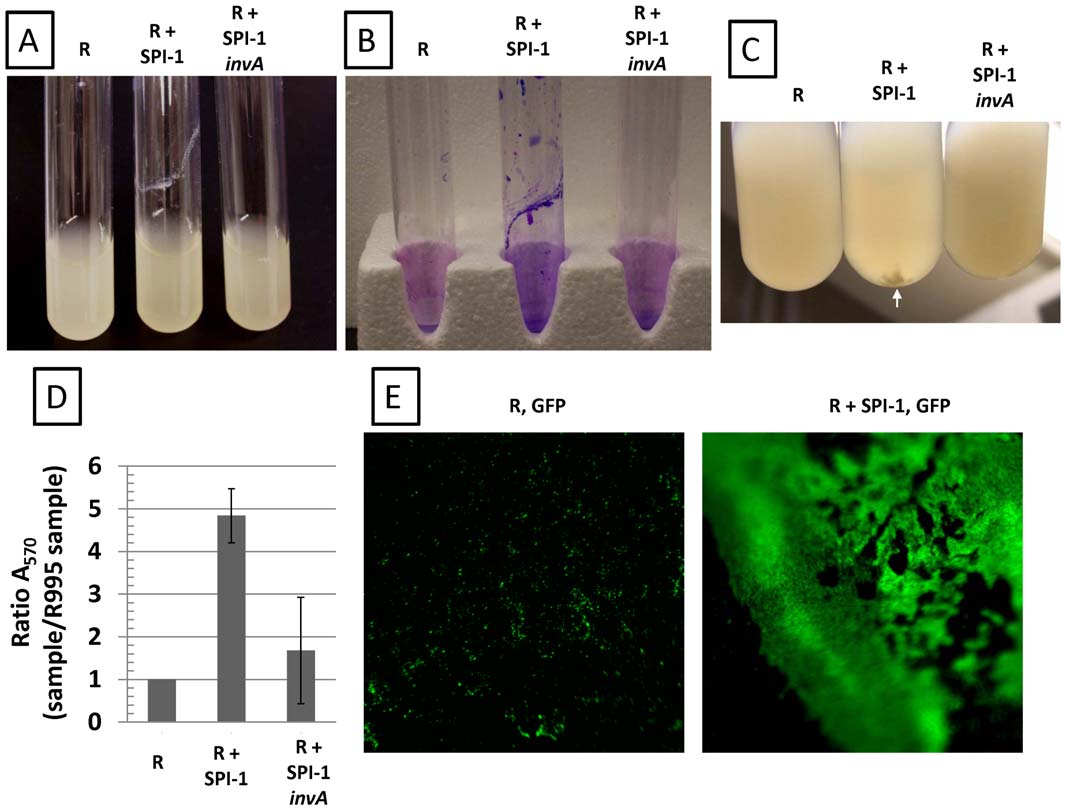
Biofilm-like cell aggregation mediated by SPI-1. **Panel A.** Cultures of the *S.* Typhimurium strain χ3339 *flhCD* containing either R995 (denoted as “R”), R995 + SPI-1, or R995 + SPI-1 *invA* are shown. Note adhered biofilm for the R995 + SPI-1 strain. **Panel B.** Crystal violet stains of cultures of the same strains as in panel A. After removal of non-adherent cells, the adherent biofilm cells were stained with crystal violet as indicated in Materials and Methods. **Panel C.** Cell clumps present in R995 + SPI-1 cultures (compared to R995 and R995 + SPI-1 *invA* cultures) are noted by the white arrow. **Panel D.** Quantification of crystal violet staining. After crystal violet staining of adhered cells and extraction of stain via acetic acid wash, the A_570_ values of extracted stains were obtained from the indicated samples. A ratio of A_570_ for each sample to the A_570_ for the R995 strain was calculated and plotted. The statistical difference between the R995 + SPI-1 and R995 + SPI-1 *invA* samples is p = 0.0004. **Panel E.** Strain χ3339 *flhCD* expressing GFP (via plasmid pGreenTIR) containing R995 or R995 + SPI-1 was grown in glass tubes, and then adhered cells were visualized after removal of the broth culture using fluorescence microscopy at the same magnification (250×). Blurriness on the edges is due to the concave nature of the culture tube. Note the presence of single cells (indicated as punctate green spots) or clumps of cells on the surface of the glass that are non-adherent in the R995, GFP sample. By contrast, in the R995 + SPI-1, GFP sample, there are a massive number of cells organized into an extensive, adhered matt on the glass surface.

In characterizing the *S.* Typhimurium strain χ3339 *flhCD* (R995 + SPI-1), we observed that broth cultures displayed a biofilm that could be stained with crystal violet (which indicates the presence of cells and is commonly used to score biofilms) (Figure 1, Panels A,B). This strain also displayed cell clumps in the media further indicating cell aggregation (Figure 1, Panel C). These phenotypes were not observed with strain χ3339 *flhCD* (R995) indicating that the presence of SPI-1 on this plasmid was responsible for the results seen with R995 + SPI-1 (Figure 1). In addition, strain χ3339 *flhCD* (R995 + SPI-1 *invA*) did not display these phenotypes as well (Figure 1). This plasmid construct is isogenic to R995 + SPI-1 but contains an insertion mutation in the *invA* gene which encodes a conserved integral membrane protein that is essential for SPI-1 type III secretion activity [15]. The observation that R995 + SPI-1 *invA* does not direct biofilm formation indicates that the presence of the chromosomal copy of *invA* (which is likely expression defective in this strain background due to the *flhCD* mutation – see below) is not sufficient to replace loss of the plasmid copy for this activity. Quantitation of crystal violet staining showed a clear statistically-significant difference between the R995 + SPI-1 and control cultures (Figure 1, Panel D). To further characterize the biofilm, we introduced a GFP-expressing plasmid into the R995 and R995 + SPI-1 strains. After culture, we used fluorescence microscopy to visualize the biofilm directly on the side of the glass tube. The R995 + SPI-1 GFP strain displayed an organized, intensely green biofilm indicating massive accumulation of cells on the side of the tube (Figure 1, Panel E). The R995 GFP strain did not form biofilm and displayed only background green fluorescence that was unorganized and mostly non-adherent single cells (Figure 1, Panel E).

### R995 + SPI-1 strains display hyper-active SPI-1 type III system functions

**Figure 2:**
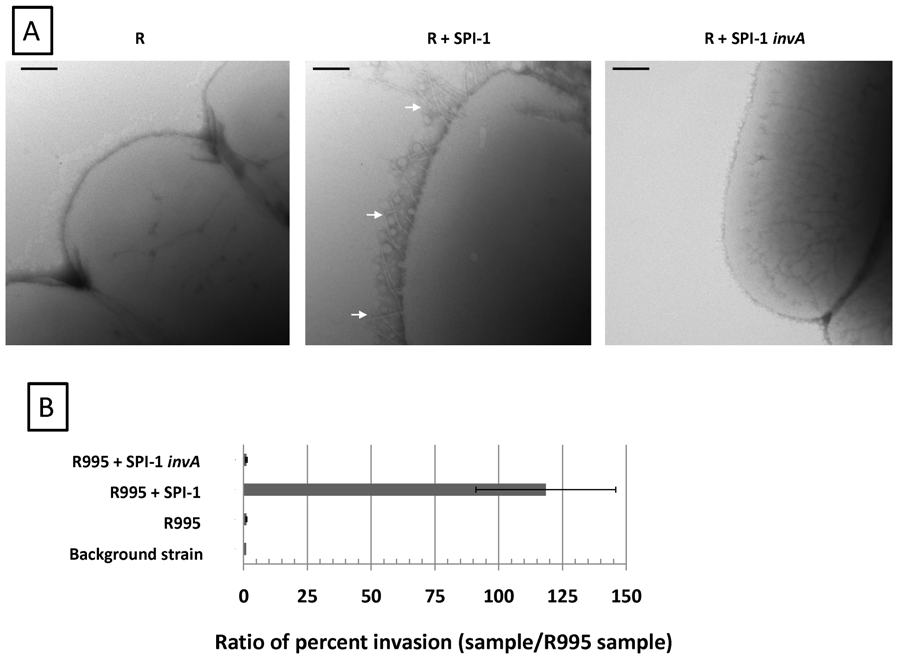
R995 + SPI-1 strains express elevated SPI-1 functions. **Panel A.** TEM micrographs of χ3339 *flhCD* strain containing R995 (denoted as “R”), R995 + SPI-1, or R995 + SPI-1 *invA*. Arrows indicate type III system needles on the surface of the R995 + SPI-1 strain. Size bar = 100 nm. **Panel B**. Comparison of invasion of indicated strains (χ3339 *flhCD* background) into Int407 intestinal epithelial cells. The ratio of percent invasion for each strain to the percent invasion for the R995 strain was calculated and plotted. Note that the *flhCD* mutation in this strain background renders these bacteria invasion defective since FlhCD plays a role in regulation of SPI-1 gene expression. Thus, an increase in invasion (indicating an elevation in SPI-1 activity expressed from R995-encoded SPI-1) is able to be easily observed in this strain background.

We hypothesized that the biofilm/cell aggregation phenotype was due to hyper-activity of the SPI-1 T3SS present on R995 + SPI-1. To determine if we could observe evidence of elevated SPI-1 activity directed by this plasmid construct, we examined *S.* Typhimurium χ3339 *flhCD* strains containing R995, R995 + SPI-1, or R995 + SPI-1 *invA* using transmission electron microscopy (Figure 2, Panel A). Previous reports have described that the type III secretion needle can be easily observed in a hyper-elongated form extending from the surface of strains that are altered in expression of key type III system components [17], [18], [19]. We found that the R995 + SPI-1 strain displayed copious amounts of elongated needles on its surface, while these needles were absent from the R995 and R995 + SPI-1 *invA* strains (Figure 2, Panel A). We also tested these strains for invasion of non-phagocytic cells, a key function that is directed by the SPI-1 type III system [12]. Note that the *flhCD* mutation in this strain background renders these bacteria invasion defective since FlhCD plays a role in regulation of chromosomal SPI-1 gene expression [20]. Thus, an increase in invasion (indicating an elevation in SPI-1 activity) is able to be easily observed in this strain background. We found that the presence of R995 + SPI-1 dramatically increased invasion to more than 100-fold above background levels seen in the R995 and R995 + SPI-1 *invA* strains (Figure 2, Panel B). Taken together, these results indicate that the biofilm/cell aggregation phenotype observed in the R995 + SPI-1 strain is associated with hyper-activity of the SPI-1 T3SS. The results also show that the R995 + SPI-1 construct is able to override the requirement of FlhCD for SPI-1 expression and function in this background.

### Characterization of the SPI-1-mediated biofilm

**Figure 3:**
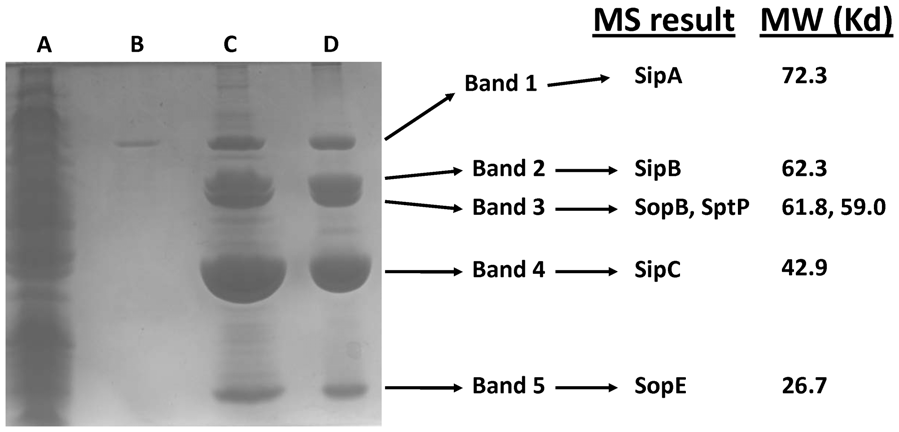
SDS-PAGE of SPI-1 biofilm. The following samples from χ3339 *flhCD* (R995 + SPI-1) culture were run in an SDS-PAGE gel and Coomassie stained: **Lane A**: total cell lysate (from approximately 0.5 ml of culture); **Lane B**: culture supernatant (from approximately 0.1 ml of culture); **Lanes C and D**: biofilms harvested from replicate samples corresponding to 2 independent cultures of the R995 + SPI-1 strain. The indicated bands were excised and identified via mass spectrometry (MS) analysis. The identification of each protein band and its corresponding molecular weight (MW) are provided.

We harvested the SP-1-mediated biofilm from the side of culture flasks, denatured the material with SDS sample buffer and heating, and ran the sample on an SDS-PAGE gel followed by Coomassie staining (Figure 3). The biofilm sample displayed massive bands at locations that were consistent with the sizes of known SPI-1 protein substrates (Figure 3). Samples consisting of SPI-1-directed cell clumps in the media (from Figure 2, Panel C) also displayed the same massive accumulation of proteins at these band sizes (data not shown). Comparison of the biofilm sample to both total cell lysate and culture supernatant indicated significant enrichment of these protein bands in the biofilm (Figure 3). Western blot analysis of the biofilms and cell clumps using antisera against SipC protein confirmed the presence of this substrate in these samples (data not shown). To determine the identity of the protein bands, we excised the bands and analyzed them via mass spectrometry. The results indicated that the bands were secreted substrates of the SPI-1 system: SipA, SipB, SipC, SopB, SopE, and SptP (Figure 3). Interestingly, these proteins consisted of substrates encoded within SPI-1 (SipA, SipB, SipC, and SptP) and outside SPI-1 in the *S.* Typhimurium chromosome (SopE and SopB).

**Figure 4:**
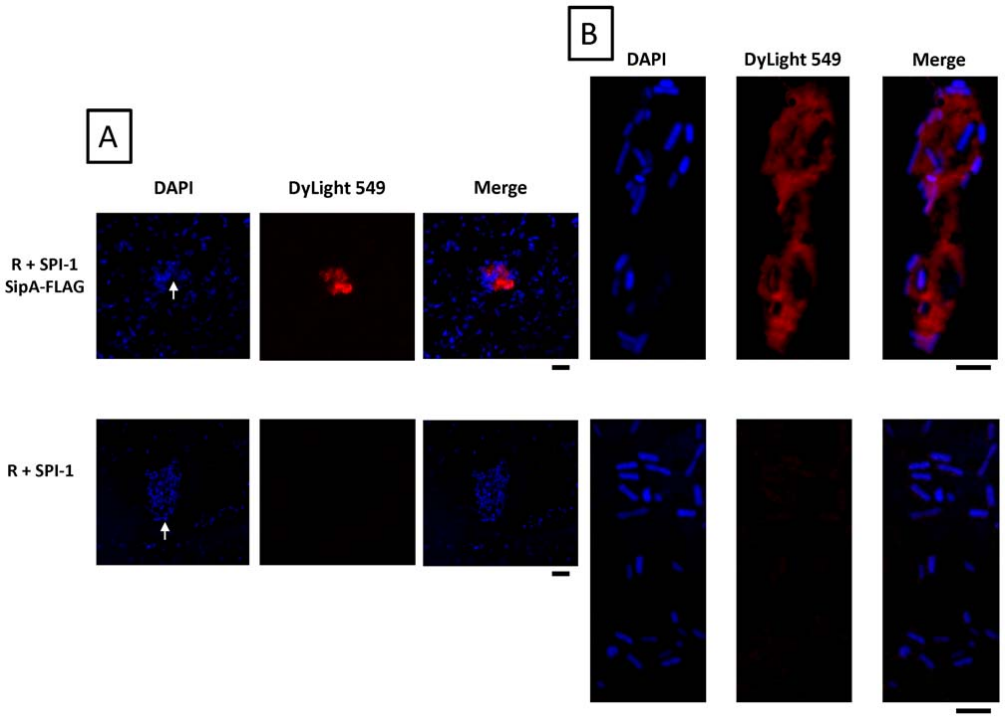
Immunofluorescence microscopy of biofilm samples. **Panel A:** Biofilms were harvested from strain χ3339 *flhCD* containing either R995 + SPI-1 SipA-FLAG or R995 + SPI-1 and visualized via immunofluorescence microscopy as described in the Results and Materials and Methods. DAPI (corresponding to cells), DyLight549 (corresponding to SipA-FLAG protein), or merged images are indicated. A cell clump from the harvested biofilm for each sample is indicated by the white arrow. Quantitation of red (DyLight549-stained) clumps for each sample is provided in Figure S2. Size bar = 3000 nm. **Panel B:** Higher magnification images of biofilm cell clumps obtained from strains as indicated in Panel A. Size bar = 2000 nm.

To further demonstrate association of SPI-1 substrate protein with the biofilm, we performed an analysis of the biofilm formed with the R995 + SPI-1 SipA-FLAG plasmid. This construct is isogenic to R995 + SPI-1 and expresses a C-terminally FLAG-tagged SipA protein [15]. This plasmid directs formation of a biofilm and cell clumps, and the presence of SipA-FLAG in the biofilm was confirmed using Western blot analysis (Figure S1). To better visualize SipA-FLAG in the biofilm, we performed immunofluorescence microscopy using antisera against the FLAG epitope. We initially attempted this experiment using glass slides or coverslips, but efforts to grow biofilms directly on these surfaces were either unsuccessful or resulted in unacceptable background. The approach that gave the best results was to grow the biofilm in a flask, wash off the vegetative cells, harvest the biofilm, transfer to a coverslip, and then process as described in Materials and Methods. For the R995 + SPI-1 SipA-FLAG samples, we observed clearly visible red clumps associated with cells indicating staining for SipA (via DyLight 549 secondary antibody) (Figure 4). These clumps consisted of bacterial cells that surround and are embedded in what appears to be a globular mass of protein (Figure 4). Clumps did not stain red for the control R995 + SPI-1 samples (Figure 4). Quantitation of these results is provided in Figure S2. Higher magnification of the R995 + SPI-1 SipA-FLAG clumps revealed unstained “halos” visible on the perimeter of many cells (Figure 4). These halos could represent a sheath-like matrix that surrounds the cells in the biofilm (see below).

**Figure 5:**
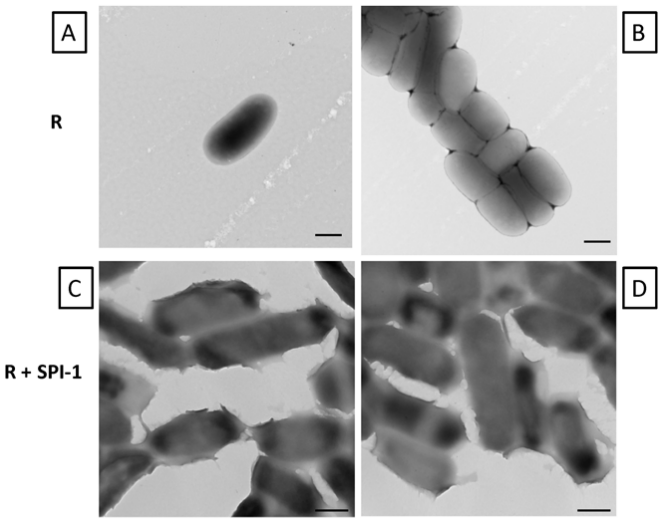
TEM analysis of samples from χ3339 *flhCD* (R995) culture and χ3339 *flhCD* (R995 + SPI-1) biofilm. **Panels A and B:** Cells from R995 culture were harvested, washed in PBS, and visualized for TEM as described in Materials and Methods. **Panels C and D:** Cells from R995 + SPI-1 biofilm were harvested and visualized for TEM as described in Materials and Methods. Note the presence of a “sheath” material on the surface of the R995 + SPI-1 biofilm cells. All size bars = 500 nm.

**Figure 6:**
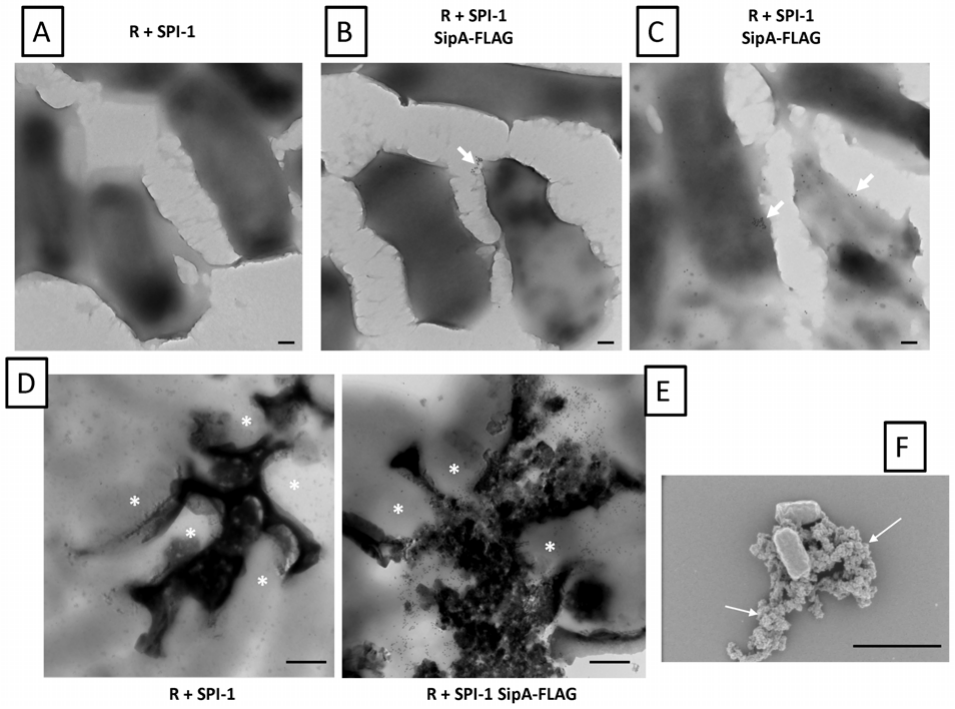
Immunogold electron microscopy and SEM analysis. Biofilm samples of strain χ3339 *flhCD* containing either R995 + SPI-1 (**Panels A and D**) or R995 + SPI-1 SipA-FLAG (**Panels B, C, and E**) were processed for immunogold electron microscopy as described in the Materials and Methods. Panels B and C are different images obtained from the same R995 + SPI-1 SipA-FLAG sample. Size bars: Panels A, B, C = 100 nm, and Panels D, E = 500 nm. Asterisks in Panels D and E indicate bacteria. Quantification of the immunogold particles for each sample is presented in Figure S4. Higher magnification images of immunogold particles from Panels B and E are shown in Figure S3. **Panel F** is an SEM image of the R995 + SPI-1 strain biofilm sample (size bar = 2000 nm). Arrows indicate extracellular, amorphous biofilm material.

We performed transmission electron microscopy on R995 + SPI-1 biofilm samples, and this revealed that the cells associated with the biofilm expressed a “sheath” material on their surface that appeared to connect individual cells (Figure 5). This sheath was not observed on cells from cultures with the R995 strain (Figure 5). We performed immunogold-electron microscopy on samples from R995 + SPI-1 and R995 + SPI-1 SipA-FLAG biofilms using antibodies against the FLAG epitope. We observed immunogold particles on the surface of the sheath for R955 + SPI-1 SipA-FLAG samples (Figure 6 and Figure S3). Immunogold particles were restricted to the surface of R995 + SPI-1 SipA-FLAG cells and were not found on the grid surface or on the surface of R995 + SPI-1 cells beyond very low background levels (Figure 6, Figure S3, and Figure S4). For the biofilm TEM samples, we also observed areas where cells were clumped together around electron-dense, amorphous, globular material that was consistent with similar observations in fluorescence microcopy samples (Figure 6, Panel D, E and Figure S3). This material (and the immediate area around this material on the cell surface) was able to be heavily punctated with immunogold particles on the R995 + SPI-1 SipA-FLAG samples (Figure 6, Panel E and Figure S3). Immunogold spots were not visible on this material in the R995 + SPI-1 samples (Figure 6, Panel D). We also performed scanning electron microscopy of R995 + SPI-1 biofilm samples and observed globular material associated with cells that was consistent with our other observations (Figure 6, Panel F). This material was not found in SEM samples using cells from R995 strain cultures (data not shown). Taken together, our data indicate that SPI-1-mediated biofilms consist of a globular extracellular material that is composed, at least in part, of copious amounts of SPI-1 substrate proteins.

### Effect of strain background, flagella, and previously identified biofilm genes

**Figure 7:**
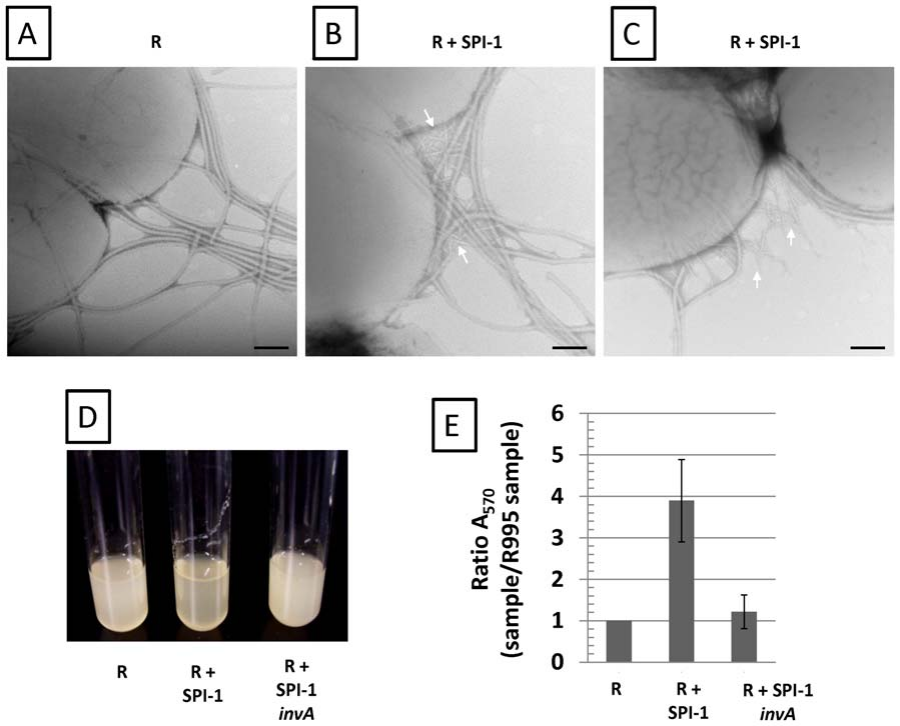
SPI-1 biofilm in flagellated strain. TEM was performed using culture samples from flagellated strain χ3339 containing R995 (**Panel A**) or R995 + SPI-1 (**Panel B and C**) (size bar = 100 nm). White arrows indicate type III secretion needles among the flagella. **Panel D**: Culture tubes containing above strains and χ3339 R995 + SPI-1 *invA* strain. Note biofilm in R995 + SPI-1- containing strain. **Panel E**: Culture tubes from the experiment in Panel D were stained with crystal violet and quantitated for staining using A_570_ values as described in the Materials and Methods. A ratio of the A_570_ value for each sample to the A_570_ value for the R995 sample was calculated and plotted. The statistical difference between the R995 + SPI-1 and R995 + SPI-1 *invA* samples is p = 0.0003.

To determine if the SPI-1-mediated biofilm could form in other *S.* Typhimurium strain backgrounds, we transferred R995 and R995 + SPI-1 to UK-1 *flhCD* and ATCC14028 *flhCD* strains and tested these strains for biofilm formation (Figure S5 and Figure S6). Biofilm was formed in the strains containing R995 + SPI-1 but not in those containing R995 (Figure S5 and Figure S6). We also observed the same result using an *S.* Typhimurium LT2 *flhCD* background (data not shown). This indicates that this phenotype is not due to a strain-specific phenomenon. To test if the presence of flagella affected SPI-1 biofilm formation, we transferred R995 and R995 + SPI-1 to the strain χ3339 which expresses flagella. We examined these strains via TEM and found that many closely-packed needles could be observed on the surface of the R995 + SPI-1 strain (among the flagella) but not on the R995 strain (Figure 7, Panels A, B, C). Biofilms were formed by the R995 + SPI-1 strain, but not by strains containing R995 or R995 + SPI-1 *invA* (Figure 7, Panels D, E). This indicates that the SPI-1 biofilm is able to form in the presence of flagella. In addition, this result indicates that WT expression of SPI-1 from its chromosomal location alone does not mediate the biofilm phenotype (since in this strain WT *flhCD* and thus WT chromosomal SPI-1 expression is present).

To determine if genes previously identified to be involved in bacterial biofilm formation play a role in the SPI-1 biofilm, we transferred R995 and R995 + SPI-1 to χ3339 *flhCD* strains containing individual mutations in *bcsA*, *csgBA*, and *bapA* and tested the resulting strains for biofilm formation (Figure S7). Previous work has demonstrated that these genes contribute to bacterial biofilm formation under certain conditions via cellulose synthesis (*bcsA*), curli fiber synthesis (*csgBA*), and expression of a large membrane protein (*bapA*) [21], [22], [23]. We found that mutations in the targeted genes did not result in loss of the ability of R995 + SPI-1 to mediate biofilm formation (Figure S7). Additional evidence supporting this result in regard to the *csgBA* mutation is that the surface appendages formed by R995 + SPI-1 strains are consistent with the morphology of known type III secretion needles and not curli fibers [24], [25], [26].

To determine if the chromosomal copy of SPI-1 is required for the biofilm phenotype observed in the presence of R995 + SPI-1, we compared biofilm formation of the strain χ3339 *flhCD* ΔSPI-1 (R995 + SPI-1) to appropriate control strains and found that this strain retained the ability to form the biofilm as observed in the background containing SPI-1 (Figure S8). In addition, this strain retained the hyper-invasion phenotype as observed with the background containing SPI-1 (Figure S8). Thus, the chromosomal copy of SPI-1 is not required for R995 + SPI-1 to form the biofilm.

### Recruitment of cells into the SPI-1 biofilm

**Figure 8:**
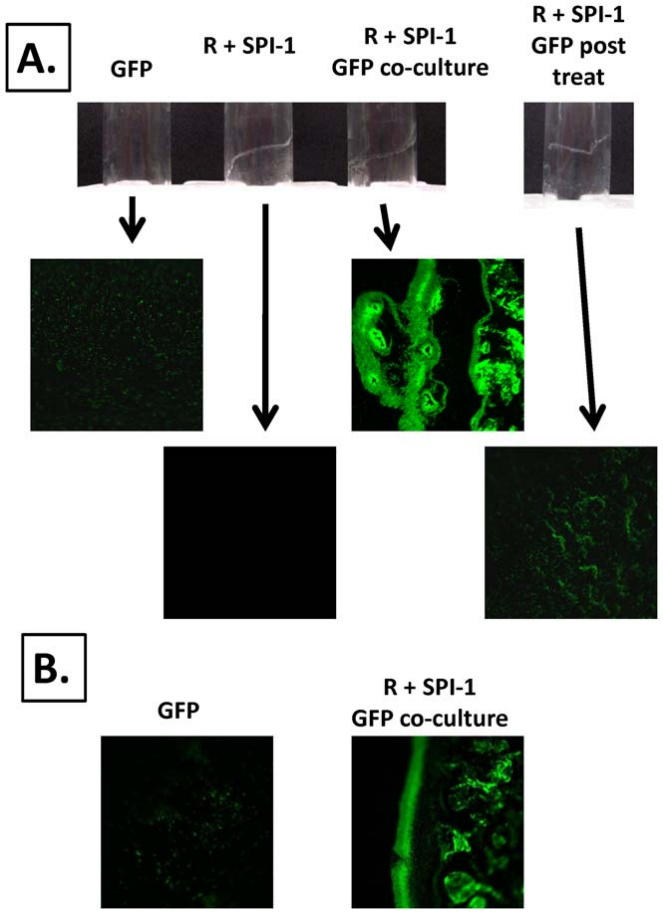
Recruitment of non-biofilm cells into SPI-1 biofilm. **Panel A:** Non-biofilm strain χ3339 *flhCD* expressing GFP and strain χ3339 *flhCD* (R995 + SPI-1) were co-cultured, and the resulting biofilm was visualized using fluorescence microscopy. Controls consisted of each strain cultured separately and biofilm post-treatment with the GFP strain as described in the text. Biofilm post-treatment consisted of exposing the surface of the pre-formed, adhered biofilm with the non-biofilm GFP strain for approximately 10 minutes followed by washing of the sample to remove non-adhered cells. This treatment is not sufficient for incorporation of the GFP strain into the organized biofilm and indicates that simultaneous growth of the two strains is required for this to occur. **Panel B**: The same experiment as in Panel A was performed, except non-biofilm *E. coli* strain ATCC25922 expressing GFP was cultured with strain χ3339 *flhCD* (R995 + SPI-1). Controls not shown gave the same results as in Panel A.

To test if bacteria that do not form biofilm could be recruited into the SPI-1 biofilm, we set up co-cultures that contained the non-biofilm strain *S.* Typhimurium χ3339 *flhCD* expressing GFP and the biofilm strain χ3339 *flhCD* (R995 + SPI-1). We also set up cultures of each strain alone. The co-culture formed a biofilm that fluoresced green as an organized adherent structure on the side of the culture tube indicating massive recruitment of the non-biofilm cells into the attached cell community (Figure 8, Panel A). The non-biofilm GFP strain alone displayed unorganized, weakly adherent fluorescence mostly representing single cells, while the R995 + SPI-1 strain alone displayed no fluorescence (Figure 8, Panel A). As an additional control, we removed planktonic cells from an R995 + SPI-1 biofilm culture and added non-biofilm GFP culture to this tube to determine if a brief exposure of the GFP cells to the biofilm allowed the same type of observation as seen with the co-culture (Figure 8, Panel A). Though some GFP cells stuck to the biofilm, this treatment did not result in the degree of organized biofilm fluorescence observed with the co-culture (Figure 8, Panel A). This suggests that at least some simultaneous growth of the strains during the co-culture is required for the non-biofilm strain to be incorporated into the biofilm as it is forming. To test if another bacterial species could be incorporated into the *S.* Typhimurium SPI-1 biofilm, we performed the same co-culture experiment with a non-biofilm *E. coli* strain expressing GFP and the biofilm strain *S.* Typhimurium χ3339 *flhCD* (R995 + SPI-1). The co-culture displayed an organized fluorescent biofilm indicating recruitment of *E. coli* into the adhered cell community, while the *E. coli* GFP strain alone displayed only background, unorganized adherence (Figure 8, Panel B). The other controls for this experiment gave the same results as seen in the experiment in Panel A (data not shown). This indicates that the recruitment of cells into the SPI-1 biofilm is not species-specific.

## Discussion

Type III secretion systems have been used for applications in bacterial engineering in which secretion and/or translocation activity can be used for beneficial purposes [27], [28], [29], [30]. The ability to study T3SS outside the context of their original genomic locations will likely lead to novel phenotypes of these systems (such as altered surface adherence, cell invasion, or protein delivery) with potentially useful applications in a range of different bacteria [15], [31], [32], [33], [34]. In this study, we report that the SPI-1 T3SS, when expressed outside its genomic context on plasmid R995, displays elevated activity which corresponds to a cell-aggregation phenotype that results in biofilm formation. Our results are consistent with a previous study that reported a pellicle phenotype associated with the *Erwinia chrysanthemi* T3SS [16]. Since two different T3SS are able to demonstrate this apparently related behavior, a key future direction of further study will be to determine if other T3SS display this activity either from their genomic location or when removed from that context. Our results indicate that WT chromosomal SPI-1, when expressed from the genomic location, does not mediate the biofilm phenotype. Moreover, we observed hyper-expression of SPI-1 phenotypes in the R995 + SPI-1 containing strains displaying biofilm. Thus, the biofilm and cell accumulation phenotypes observed here are correlated with hyper-activity of the SPI-1 T3SS. It is worth noting that co-regulation of expression of genes required for type III secretion and biofilm formation has been extensively described in *Pseudomonas aeruginosa* [35], [36], [37], [38], [39]. The proteins RetS and LadS (which are sensor kinases) and FimL (which regulates cAMP levels) control pathways that regulate expression of both type III secretion and biofilm genes in *P. aeruginosa* [36], [38], [39], [40]. Small RNAs *rsmY* and *rsmZ* are involved in the RetS pathway for this co-regulation with *rsmZ* forming a convergence point at which the RetS and LadS pathways both function in this regulatory scheme [36], [38]. Mutation or repression of type III secretion genes in *P. aeruginosa* results in enhanced biofilm in this species [35], [37]. In addition, microarray analysis of the *P. aeruginosa* cystic fibrosis epidemic strain AES-2 demonstrated up-regulated expression of type III secretion genes in biofilms formed by this strain [41]. Relating such pathways and phenotypic observations to the formation of type III secretion-mediated biofilms in *S.* Typhimurium, *P. aeruginosa*, or other species could form the basis of future work.

Our data indicate that multiple SPI-1 protein substrates are enriched for and present in the biofilm: SipA, SipB, SipC, SopB, SopE, and SptP. In addition, microscopy indicates that the biofilm is associated with a globular, amorphous extracellular material that is able to be labeled using an epitope-tagged SipA protein. Bacterial cells appear to be embedded in this globular material, and a sheath-like coating appears to be present on cells associated with the biofilm. We do not know if SPI-1 substrates or other materials are forming this sheath coating, but we did observe labeling of the sheath in the R995 + SPI-1 SipA-FLAG samples in our immunogold electron microscopy analysis. Our data seem to provide more evidence for the globular extracellular material being composed of SipA as compared to the sheath (combining observations from immunofluorescence and immunogold electron microscopy). However, the sheath could be composed of other SPI-1 substrates in addition to SipA. The sheath could also be composed of other materials that are induced upon biofilm formation or cell aggregation, such as polysaccharide or other proteins. However, our data suggest that any polysaccharide component would be synthesized independently of the *bcsA* gene involved in the cellulose pathway. Further experiments will be needed to more fully characterize the relationship of SPI-1 biofilm surface structures and to understand the contribution of individual SPI-1 substrate proteins to biofilm formation. The identification of SipB and SipC in biofilm samples is consistent with previous studies that implicate these proteins in the adherence of *S.* Typhimurium to tissue culture cells [42]. In addition, the SipC protein has been shown to multimerize and could form structures that are involved in the biofilm formation and bacterial aggregation observed here (however this activity would likely require the function of other SPI-1 secreted proteins to form the overall biofilm structure) [6], [43]. Moreover, purified SipA protein possesses biochemical activity involving cell surface interactions, and it is possible that this activity could contribute to the role of SipA in the formation of biofilm or cell aggregates [44]. The copious amount of SP-1 proteins that are harvested from the biofilm and cell clumps could be potentially easily purified (using an epitope tag, SDS-PAGE separation, or other approaches) and used for other purposes such as vaccination, protein injection experiments, or other biochemical assays.

The SPI-1 biofilm formed in multiple *S.* Typhimurium backgrounds, in the presence of flagella, and in the absence of genes previously shown to be required for known biofilm pathways. This indicates the potential for different kinds of bacteria to express the SPI-1 biofilm phenotype. However, our efforts to test for SPI-1 biofilm formation in other bacterial species containing R995 + SPI-1 are hampered by the fact that when SPI-1 is transferred to other Gram negative species, it displays a gene expression defect [15]. We currently do not know why this defect occurs, and we are pursuing methods to study and ultimately overcome this issue. This will lead to a greater understanding of how the regulation of SPI-1 gene expression evolved and how it can be engineered in other Gram negative bacteria. The possible reasons for defective SPI-1 expression in other Gram negative species could include the absence of one or more key SPI-1 gene regulators in other Gram negative species or the presence of mechanisms that direct repression or degradation of SPI-1 transcripts or proteins in these species (which are absent in *S.* Typhimurium). Alternatively, prokaryotic histone-like proteins that form DNA nucleoid architecture (such as HNS) may act differently on R995 + SPI-1 in other Gram negative species such that they abnormally repress SPI-1 expression in these backgrounds.

Non-biofilm *S.* Typhimurium and *E. coli* cells were able to be recruited into the SPI-1 biofilm. This has significant implications for engineered biofilm formation using this system for situations where adhered heterologous bacterial communities are desired. There are several potential uses for engineered biofilm formation including bioremediation, biofuel cell design, and engineered infections for beneficial purposes [45], [46], [47]. In relation to this point, we note that the SPI-1 biofilm was able to be observed in culture using minimal media (data not shown). The ability to use the SPI-1 T3SS or other T3SS for these applications is an area of study that is able to be potentially developed and may include modification to the T3SS to mitigate undesired transfer or side effects of the genes. In addition, the results presented here indicate the potential in studying T3SS outside the context of their original genomic locations to yield previously undiscovered phenotypes that may impact our ability to engineer bacteria.

## Materials and Methods

### Ethics Statement

The Int-407 intestinal epithelial cell line was obtained from Dr. Cheryl Nickerson [48].

### Strains, plasmids, and growth media

For a list of the strains and plasmids used in this study, please refer to Table 1 [15], [49], [50], [51]. The non-flagellated strain χ3339 *flhCD* was constructed in the χ3339 background using the lambda Red system as described previously with primers designed to delete the adjacent *flhC* and *flhD* open reading frames [52]. This mutation was transferred to the UK-1 and ATCC14028 backgrounds using standard P22 transduction as described previously [53]. The *bcsA*, *csgBA*, and *bapA* mutations were constructed similarly using lambda Red recombination [52]. All primers used for making the chromosomal gene deletions are included in Table S1. Strains were grown in Lennox broth (supplemented with additional NaCl to 0.3 M final concentration) shaking at 37 degrees Celsius in glass tubes or flasks. All studies were performed in accordance with established biosafety guidelines as described in the NIH Guidelines for Research Involving Recombinant DNA Molecules.

**Table 1:** Strains and plasmids used in this study.

Strain/Plasmid	Comments
κ3339	*Salmonella* Typhimurium, reference 49
κ3339 *flhCD*	*Salmonella* Typhimurium, this study
κ3339 *bcsA*	*Salmonella* Typhimurium, this study
κ3339 *bapA*	*Salmonella* Typhimurium, this study
κ3339 *csgBA*	*Salmonella* Typhimurium, this study
UK-1 *flhCD*	*Salmonella* Typhimurium, this study
ATCC14028 *flhCD*	*Salmonella* Typhimurium, this study
ATCC25922	*Escherichia coli*, American Type Culture Collection
R995	Reference 51
R995 + SPI-1	Reference 15
R995 + SPI-1 *invA*	Reference 15
R995 + SPI-1 SipA-FLAG	Reference 15
pGreenTIR	Reference 50

### Biofilm assays

Biofilms grown in either glass tubes or Erlenmeyer flasks were stained with crystal violet and quantified via acetic acid wash and absorbance at 570 nm as described previously [54]. The results of crystal violet quantification were obtained from four to eight individual cultures for each strain and ranged from 0.03–2.5 in A_570_ values. To harvest biofilms, media containing planktonic bacteria were removed, flasks were washed with PBS, and the biofilms were removed by scraping into a small amount of PBS (100–200 microliters). Cell clumps in the media were removed by pipetting or with an inoculating loop and transferred to a fresh microfuge tube. SDS sample buffer was added to the biofilm or cell clump samples for SDS-PAGE analysis, Coomassie staining, and Western blotting using previously described protocols [15], [55]. Individual bands were excised from a Coomassie stained gel for mass spectrometry (Alphalyse, Inc., Palo Alto, CA). To test for recruitment of cells into the biofilm, non-biofilm strains *S.* Typhimurium strain χ3339 *flhCD* or *Escherichia coli* ATCC25922 containing pGreenTIR were co-inoculated with *S.* Typhimurium Δ3339 *flhCD* (R995 + SPI-1) from fresh plates into broth cultures. Control cultures containing each strain alone were also inoculated, and all cultures were grown overnight as described above. Biofilms were visualized via microscopy as described below after removal of planktonic cells. For the GFP post-treatment control, planktonic cells were removed from an R995 + SPI-1 culture, non-biofilm GFP+ planktonic cultures were added to this tube, and the tube was incubated with slight agitation for 10 minutes. The planktonic cells were then removed before microscopy.

### Microscopy

Transmission and scanning electron microscopy (TEM and SEM, respectively) were performed using standard protocols as previously described except as noted below [19], [56], [57]. The images shown are between 10,000–50,000× magnification. For TEM, carbon-coated copper grids (300 mesh) were used, cells were negatively stained using 2% PTA (pH = 7.0), and samples were analyzed using an Hitachi H-7600 TEM microscope at 80 kV. For SEM, samples were sequentially-dehydrated using ethanol and subjected to critical point drying using a Polaron device before analysis using an Hitachi S-4800 SEM microscope. For immunogold electron microscopy, samples were processed as described previously except that the grids were pre-blocked using 5% BSA in PBS for 15 minutes before adding the sample [58]. Antibodies used were affinity-purified rabbit anti-FLAG (Immunology Consultants Laboratory, Newberg, OR) and colloidal gold (12 nm) affinity-purified donkey anti-rabbit IgG (Jackson Immunoresearch, West Grove, PA). For immunofluorescence microscopy, samples were processed as described previously [42] except that biofilms were harvested from the side of a flask into 200 ul PBS before processing, and affinity-purified rabbit anti-FLAG (as above) and DyLight 549-conjugated affinity-purified donkey anti-rabbit IgG (Jackson Immunoresearch, West Grove, PA) were used. An Olympus IX70 fluorescence microscope was used to visualize samples. For visualizing GFP biofilms, planktonic cells were removed from cultures grown in glass tubes followed by a brief wash of the tubes with PBS (to remove non-adherent planktonic cells) and direct examination via fluorescence microscopy.

### Invasion assay

To test indicated strains for invasion of Int407 intestinal epithelial cells, a standard gentimicin protection assay was performed as described previously [15], [59]. For these assays, the *S.* Typhimurium χ3339 *flhCD* background was used, and this strain has a previously reported invasion defect due to involvement of FlhCD in SPI-1 gene expression [20]. Thus, the greater than 100-fold invasion increase due to the presence of R995 + SPI-1 in Figure 2 is able to be observed in this strain background. The results are obtained from four to seven individual experiments each plated in triplicate.

## Supporting Information

Figure S1 **Biofilm of R995 + SPI-1 SipA-FLAG.** **Panel A:** Cultures of strain χ3339 *flhCD* containing either R995 or R995 + SPI-1 SipA-FLAG. **Panel B:** Western blot probed with anti-FLAG antibody such that the protein band indicates SipA-FLAG. The strain background is χ3339 *flhCD*. Lane 1: total cell lysate, R995 + SPI-1; Lane 2: total cell lysate, R995 + SPI-1 SipA-FLAG; Lane 3: biofilm, R995 + SPI-1; Lanes 4 and 5: replicate biofilm samples, R995 + SPI-1 SipA-FLAG.
(PDF)

Figure S2 **Quantification of red (DyLight 549) cell clumps observed per 2300 square micrometers field of view via immunofluorescence microscopy of biofilm samples.** Strains are χ3339 *flhCD* containing either R995 + SPI-1 or R995 + SPI-1 SipA-FLAG. Cultures were grown for biofilm formation, and then the biofilms were harvested and transferred to a coverslip for fluorescence microscopy as described in Materials and Methods. Biofilm cell clumps stained positively for SipA-FLAG (DyLight 549) were quantified. At least nine different fields of view were used per sample.
(PDF)

Figure S3 **Higher magnification of immunogold particles on the surface of strain χ3339** ***flhCD*** **(R995 + SPI-1 SipA-FLAG).** Panel A: Image from Figure 6, Panel B in text. Panel B: Image from Figure 6, Panel E in text.
(PDF)

Figure S4 **Quantification of immunogold particles observed per 9 square micrometers field of view via TEM of biofilm samples.** Strains are χ3339 *flhCD* containing either R995 + SPI-1 or R995 + SPI-1 SipA-FLAG. Cultures were grown for biofilm formation, and then the biofilms were harvested and transferred to a grid for transmission electron microscopy as described in Materials and Methods. The number of immunogold particles per 9 square micrometers field of view were quantified. At least sixteen different fields of view were used per sample.
(PDF)

Figure S5 **SPI-1 biofilms in** ***S.*** **Typhimurium UK-1** ***flhCD*** **and ATCC14028** ***flhCD*** **backgrounds.** **Panel A:** Culture flasks of strain UK-1 *flhCD* containing either R995 or R995 + SPI-1. **Panel B:** Culture flasks of strain ATCC14028 *flhCD* containing either R995 or R995 + SPI-1. The white arrow indicates biofilm present in the R995 + SPI-1 cultures.
(PDF)

Figure S6 **Quantification of crystal violet staining of SPI-1 biofilms in** ***S.*** **Typhimurium UK-1** ***flhCD*** **and ATCC14028** ***flhCD*** **backgrounds.** Cultures as described in Figure S5 were processed for crystal violet staining as in the Materials and Methods. The A_570_ values were obtained from stained samples, and a ratio of A_570_ for each sample to the A_570_ for the R995 strain was calculated and plotted. The statistical difference between the R995 and R995 + SPI-1 samples for each strain was p = 0.0032 (UK-1 *flhCD*) and p = 0.0036 (ATCC14028 *flhCD*). At least four independent cultures were assayed for each strain.
(PDF)

Figure S7 **SPI-1 biofilms in** ***S.*** **Typhimurium χ3339** ***flhCD*** **strains containing mutations in** ***bcsA*** **,** ***bapA*** **, and** ***csgBA*** **.** **Panel A.** Cultures of the *S.* Typhimurium strain χ3339 *flhCD* containing mutations in *bcsA*, *bapA*, or *csgBA* and containing the plasmids R995 (denoted as “R”) or R995 + SPI-1 are shown. Note adhered biofilm for the R995 + SPI-1 strains indicated by white arrows. **Panel B.** Quantification of crystal violet staining. Cultures as described in panel A were processed for crystal violet staining as in the Materials and Methods. The A_570_ values were obtained from stained samples, and a ratio of A_570_ for each sample to the A_570_ for the R995 strain was calculated and plotted. The statistical difference between the R995 and R995 + SPI-1 samples for each mutant strain was p = 0.01 (*bcsA*), p = 0.0042 (*bapA*), and p = 0.004 (*csgBA*). At least four independent cultures were assayed for each strain.
(PDF)

Figure S8 **R995 + SPI-1 displays hyper-invasion and biofilm phenotypes in the absence of chromosomal SPI-1.** **Panel A**: *S.* Typhimurium strain χ3339 *flhCD* ΔSPI-1 containing R995 + SPI-1 was tested for Int407 cell invasion compared to isogenic control strains containing either no plasmid or plasmid R995. The percent invasion of each strain (based on initial inoculum) was calculated. The data is presented as a ratio of the percent invasion for each strain to the percent invasion of the R995 strain. Note that the background strain is non-invasive due to the ΔSPI-1 mutation. **Panel B**: Cultures of strain χ3339 *flhCD* ΔSPI-1 containing either R995 or R995 + SPI-1 were processed for crystal violet biofilm staining as in the Materials and Methods. The A_570_ values were obtained from stained samples, and a ratio of A_570_ for each sample to the A_570_ for the R995 strain was calculated and plotted. The statistical difference between the R995 and R995 + SPI-1 samples was p = 0.001. Ten independent cultures were assayed for each strain.
(PDF)

Table S1 **Primers used to construct mutations using lambda Red recombination.**
(DOC)

## References

[1] Cornelis GR (2006). The type III secretion injectisome.. Nat Rev Microbiol.

[2] Galan JE, Wolf-Watz H (2006). Protein delivery into eukaryotic cells by type III secretion machines.. Nature.

[3] Wagner S, Konigsmaier L, Lara-Tejero M, Lefebre M, Marlovits TC (2010). Organization and coordinated assembly of the type III secretion export apparatus.. Proc Natl Acad Sci U S A.

[4] Hersh D, Monack DM, Smith MR, Ghori N, Falkow S (1999). The Salmonella invasin SipB induces macrophage apoptosis by binding to caspase-1.. Proc Natl Acad Sci U S A.

[5] Mueller CA, Broz P, Cornelis GR (2008). The type III secretion system tip complex and translocon.. Mol Microbiol.

[6] Myeni SK, Zhou D (2010). The C terminus of SipC binds and bundles F-actin to promote Salmonella invasion.. J Biol Chem.

[7] Galan JE (2009). Common themes in the design and function of bacterial effectors.. Cell Host Microbe.

[8] McGhie EJ, Brawn LC, Hume PJ, Humphreys D, Koronakis V (2009). Salmonella takes control: effector-driven manipulation of the host.. Curr Opin Microbiol.

[9] Waterman SR, Holden DW (2003). Functions and effectors of the Salmonella pathogenicity island 2 type III secretion system.. Cell Microbiol.

[10] Campellone KG, Leong JM (2003). Tails of two Tirs: actin pedestal formation by enteropathogenic E. coli and enterohemorrhagic E. coli O157:H7.. Curr Opin Microbiol.

[11] Valdez Y, Ferreira RB, Finlay BB (2009). Molecular mechanisms of Salmonella virulence and host resistance.. Curr Top Microbiol Immunol.

[12] Zhou D, Galan J (2001). Salmonella entry into host cells: the work in concert of type III secreted effector proteins.. Microbes Infect.

[13] Porwollik S, Wong RM, McClelland M (2002). Evolutionary genomics of Salmonella: gene acquisitions revealed by microarray analysis.. Proc Natl Acad Sci U S A.

[14] Collazo CM, Galan JE (1997). The invasion-associated type III system of Salmonella Typhimurium directs the translocation of Sip proteins into the host cell.. Mol Microbiol.

[15] Wilson JW, Nickerson CA (2006). Cloning of a functional Salmonella SPI-1 type III secretion system and development of a method to create mutations and epitope fusions in the cloned genes.. J Biotechnol.

[16] Yap MN, Yang CH, Barak JD, Jahn CE, Charkowski AO (2005). The Erwinia chrysanthemi type III secretion system is required for multicellular behavior.. J Bacteriol.

[17] Kubori T, Sukhan A, Aizawa SI, Galan JE (2000). Molecular characterization and assembly of the needle complex of the Salmonella Typhimurium type III protein secretion system.. Proc Natl Acad Sci U S A.

[18] Marlovits TC, Kubori T, Lara-Tejero M, Thomas D, Unger VM (2006). Assembly of the inner rod determines needle length in the type III secretion injectisome.. Nature.

[19] Tamano K, Katayama E, Toyotome T, Sasakawa C (2002). Shigella Spa32 is an essential secretory protein for functional type III secretion machinery and uniformity of its needle length.. J Bacteriol.

[20] Eichelberg K, Galan JE (2000). The flagellar sigma factor FliA (sigma(28)) regulates the expression of Salmonella genes associated with the centisome 63 type III secretion system.. Infect Immun.

[21] Barnhart MM, Chapman MR (2006). Curli biogenesis and function.. Annu Rev Microbiol.

[22] Latasa C, Roux A, Toledo-Arana A, Ghigo JM, Gamazo C (2005). BapA, a large secreted protein required for biofilm formation and host colonization of Salmonella enterica serovar Enteritidis.. Mol Microbiol.

[23] Solano C, Garcia B, Valle J, Berasain C, Ghigo JM (2002). Genetic analysis of Salmonella enteritidis biofilm formation: critical role of cellulose.. Mol Microbiol.

[24] Epstein EA, Reizian MA, Chapman MR (2009). Spatial clustering of the curlin secretion lipoprotein requires curli fiber assembly.. J Bacteriol.

[25] Hammer ND, Schmidt JC, Chapman MR (2007). The curli nucleator protein, CsgB, contains an amyloidogenic domain that directs CsgA polymerization.. Proc Natl Acad Sci U S A.

[26] Wang X, Hammer ND, Chapman MR (2008). The molecular basis of functional bacterial amyloid polymerization and nucleation.. J Biol Chem.

[27] Crepin VF, Shaw R, Knutton S, Frankel G (2005). Molecular basis of antigenic polymorphism of EspA filaments: development of a peptide display technology.. J Mol Biol.

[28] Li CM, Hienonen E, Haapalainen M, Kontinen VP, Romantschuk M (2007). Type III secretion system-associated pilus of Pseudomonas syringae as an epitope display tool.. FEMS Microbiol Lett.

[29] Panthel K, Meinel KM, Sevil Domenech VE, Trulzsch K, Russmann H (2008). Salmonella type III-mediated heterologous antigen delivery: a versatile oral vaccination strategy to induce cellular immunity against infectious agents and tumors.. Int J Med Microbiol.

[30] Xiong G, Husseiny MI, Song L, Erdreich-Epstein A, Shackleford GM (2010). Novel cancer vaccine based on genes of Salmonella pathogenicity island 2.. Int J Cancer.

[31] Ham JH, Bauer DW, Fouts DE, Collmer A (1998). A cloned Erwinia chrysanthemi Hrp (type III protein secretion) system functions in Escherichia coli to deliver Pseudomonas syringae Avr signals to plant cells and to secrete Avr proteins in culture.. Proc Natl Acad Sci U S A.

[32] Hansen-Wester I, Chakravortty D, Hensel M (2004). Functional transfer of Salmonella pathogenicity island 2 to Salmonella bongori and Escherichia coli.. Infect Immun.

[33] McDaniel TK, Kaper JB (1997). A cloned pathogenicity island from enteropathogenic Escherichia coli confers the attaching and effacing phenotype on E. coli K-12.. Mol Microbiol.

[34] Wilson JW, Coleman C, Nickerson CA (2007). Cloning and transfer of the Salmonella pathogenicity island 2 type III secretion system for studies of a range of gram-negative genera.. Appl Environ Microbiol.

[35] Atkinson S, Goldstone RJ, Joshua GW, Chang CY, Patrick HL (2011). Biofilm development on Caenorhabditis elegans by Yersinia is facilitated by quorum sensing-dependent repression of type III secretion.. PLoS Pathog.

[36] Bordi C, Lamy MC, Ventre I, Termine E, Hachani A (2010). Regulatory RNAs and the HptB/RetS signalling pathways fine-tune Pseudomonas aeruginosa pathogenesis.. Mol Microbiol.

[37] Kuchma SL, Connolly JP, O'Toole GA (2005). A three-component regulatory system regulates biofilm maturation and type III secretion in Pseudomonas aeruginosa.. J Bacteriol.

[38] Ventre I, Goodman AL, Vallet-Gely I, Vasseur P, Soscia C (2006). Multiple sensors control reciprocal expression of Pseudomonas aeruginosa regulatory RNA and virulence genes.. Proc Natl Acad Sci U S A.

[39] Whitchurch CB, Beatson SA, Comolli JC, Jakobsen T, Sargent JL (2005). Pseudomonas aeruginosa fimL regulates multiple virulence functions by intersecting with Vfr-modulated pathways.. Mol Microbiol.

[40] Inclan YF, Huseby MJ, Engel JN (2011). FimL regulates cAMP synthesis in Pseudomonas aeruginosa.. PLoS One.

[41] Manos J, Arthur J, Rose B, Bell S, Tingpej P (2009). Gene expression characteristics of a cystic fibrosis epidemic strain of Pseudomonas aeruginosa during biofilm and planktonic growth.. FEMS Microbiol Lett.

[42] Lara-Tejero M, Galan JE (2009). Salmonella enterica serovar Typhimurium pathogenicity island 1-encoded type III secretion system translocases mediate intimate attachment to nonphagocytic cells.. Infect Immun.

[43] Chang J, Myeni SK, Lin TL, Wu CC, Staiger CJ (2007). SipC multimerization promotes actin nucleation and contributes to Salmonella-induced inflammation.. Mol Microbiol.

[44] Lee CA, Silva M, Siber AM, Kelly AJ, Galyov E (2000). A secreted Salmonella protein induces a proinflammatory response in epithelial cells, which promotes neutrophil migration.. Proc Natl Acad Sci U S A.

[45] Read ST, Dutta P, Bond PL, Keller J, Rabaey K (2010). Initial development and structure of biofilms on microbial fuel cell anodes.. BMC Microbiol.

[46] Singh R, Paul D, Jain RK (2006). Biofilms: implications in bioremediation.. Trends Microbiol.

[47] Wood TK, Hong SH, Ma Q (2011). Engineering biofilm formation and dispersal.. Trends Biotechnol.

[48] Nickerson CA, Goodwin TJ, Terlonge J, Ott CM, Buchanan KL (2001). Three-dimensional tissue assemblies: novel models for the study of Salmonella enterica serovar Typhimurium pathogenesis.. Infect Immun.

[49] Gulig PA, Curtiss R (1987). Plasmid-associated virulence of Salmonella Typhimurium.. Infect Immun.

[50] Miller WG, Lindow SE (1997). An improved GFP cloning cassette designed for prokaryotic transcriptional fusions.. Gene.

[51] Wilson JW, Figurski DH, Nickerson CA (2004). VEX-capture: a new technique that allows in vivo excision, cloning, and broad-host-range transfer of large bacterial genomic DNA segments.. J Microbiol Methods.

[52] Datsenko KA, Wanner BL (2000). One-step inactivation of chromosomal genes in Escherichia coli K-12 using PCR products.. Proc Natl Acad Sci U S A.

[53] Sternberg NL, Maurer R (1991). Bacteriophage-mediated generalized transduction in Escherichia coli and Salmonella Typhimurium.. Methods Enzymol.

[54] Crawford RW, Reeve KE, Gunn JS (2010). Flagellated but not hyperfimbriated Salmonella enterica serovar Typhimurium attaches to and forms biofilms on cholesterol-coated surfaces.. J Bacteriol.

[55] Ausubel F al. e (1996). Current Protocols in Molecular Biology.

[56] Chakravortty D, Rohde M, Jager L, Deiwick J, Hensel M (2005). Formation of a novel surface structure encoded by Salmonella Pathogenicity Island 2.. EMBO J.

[57] Kubori T, Matsushima Y, Nakamura D, Uralil J, Lara-Tejero M (1998). Supramolecular structure of the Salmonella Typhimurium type III protein secretion system.. Science.

[58] Nallapareddy SR, Singh KV, Sillanpaa J, Garsin DA, Hook M (2006). Endocarditis and biofilm-associated pili of Enterococcus faecalis.. J Clin Invest.

[59] Wilson JW, Nickerson CA (2006). A new experimental approach for studying bacterial genomic island evolution identifies island genes with bacterial host-specific expression patterns.. BMC Evol Biol.

