# Supplementary figures and images for: Characterization of Salmonella Type III Secretion Hyper-Activity Which Results in Biofilm-Like Cell Aggregation
Source: PLoS One. 2012 Mar 8;7(3):e33080. doi: 10.1371/journal.pone.0033080 (PMC3297627; doi:10.1371/journal.pone.0033080)

**Figure S1.**

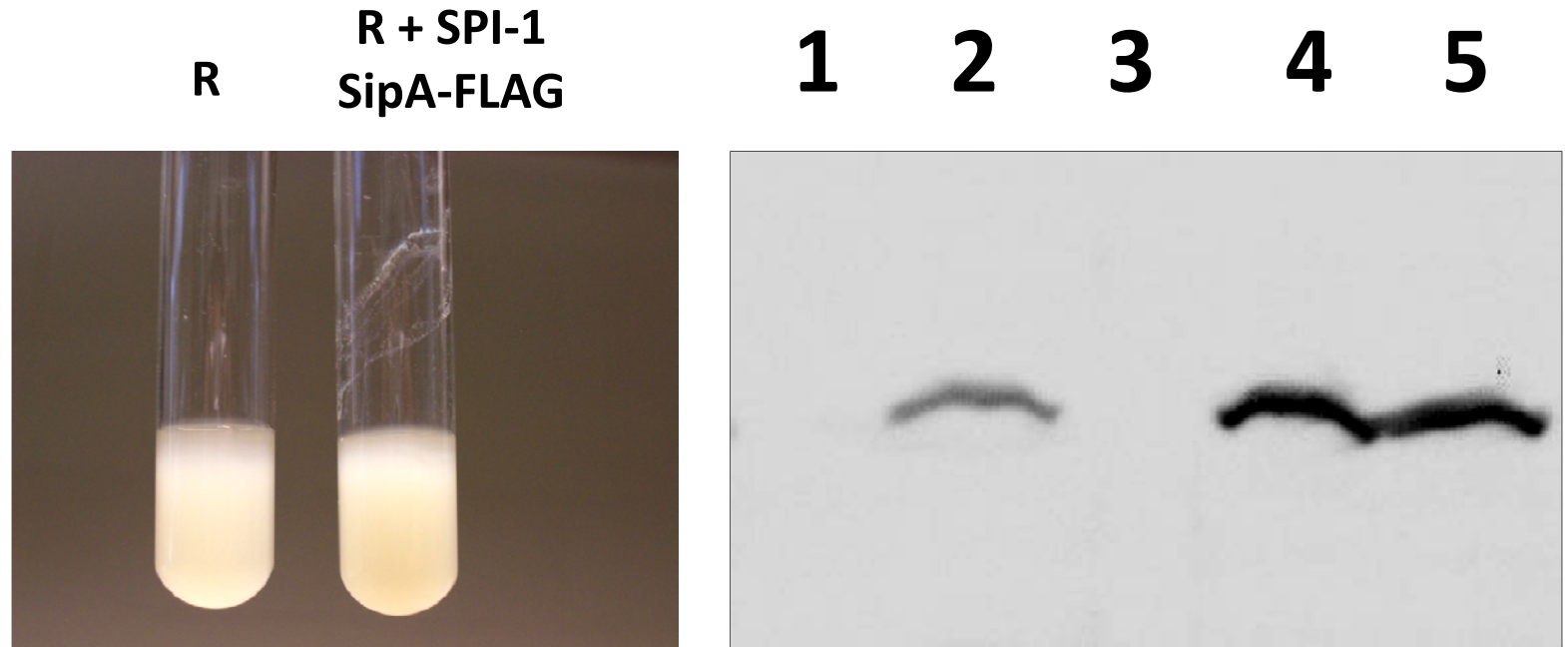

Supplement: Figure S1 — Biofilm of R995 + SPI-1 SipA-FLAG. Panel A: Cultures of strain χ3339 flhCD containing either R995 or R995 + SPI-1 SipA-FLAG. Panel B: Western blot probed with anti-FLAG antibody such that the protein band indicates SipA-FLAG. The strain background is χ3339 flhCD. Lane 1: total cell lysate, R995 + SPI-1; Lane 2: total cell lysate, R995 + SPI-1 SipA-FLAG; Lane 3: biofilm, R995 + SPI-1; Lanes 4 and 5: replicate biofilm samples, R995 + SPI-1 SipA-FLAG. (PDF) [file pone.0033080.s001.pdf]

**Figure S2.**

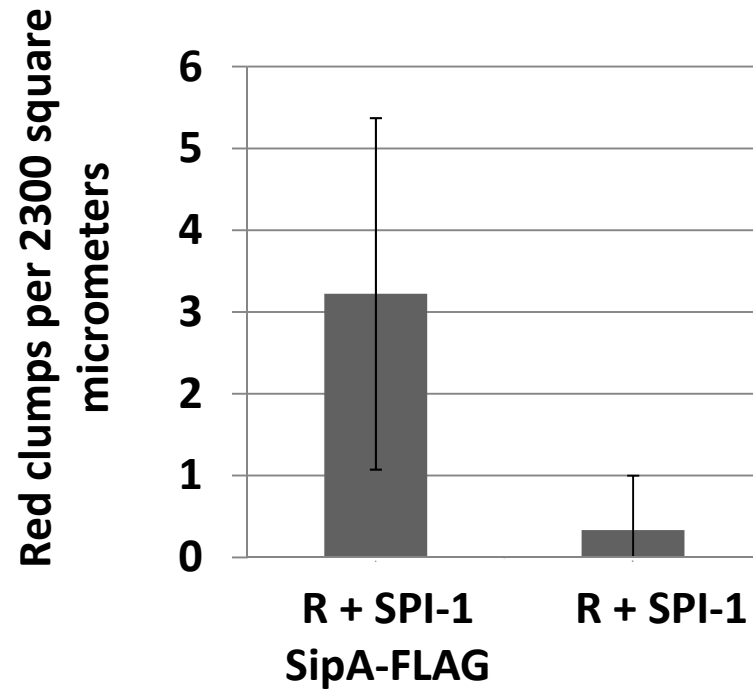

p-value = 0.0044

Supplement: Figure S2 — Quantification of red (DyLight 549) cell clumps observed per 2300 square micrometers field of view via immunofluorescence microscopy of biofilm samples. Strains are χ3339 flhCD containing either R995 + SPI-1 or R995 + SPI-1 SipA-FLAG. Cultures were grown for biofilm formation, and then the biofilms were harvested and transferred to a coverslip for fluorescence microscopy as described in Materials and Methods. Biofilm cell clumps stained positively for SipA-FLAG (DyLight 549) were quantified. At least nine different fields of view were used per sample. (PDF) [file pone.0033080.s002.pdf]

**Figure S3.**

**A**

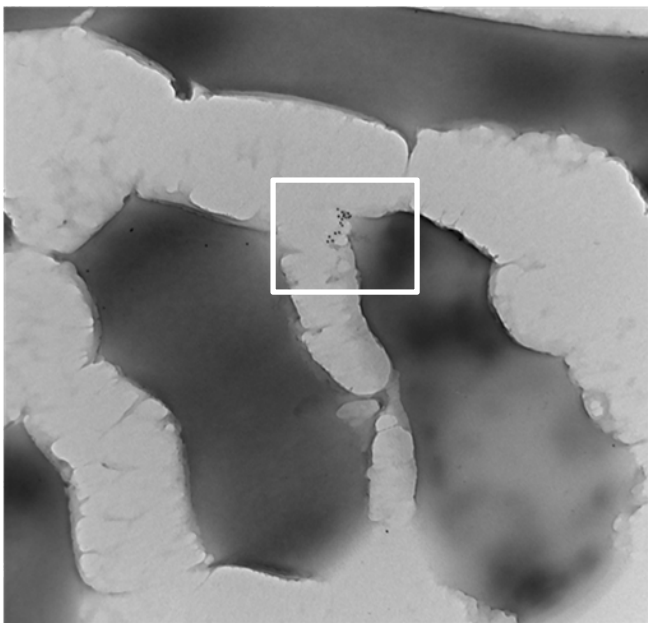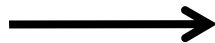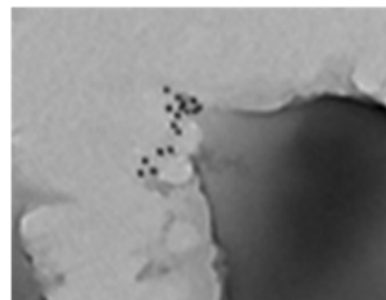

**B**

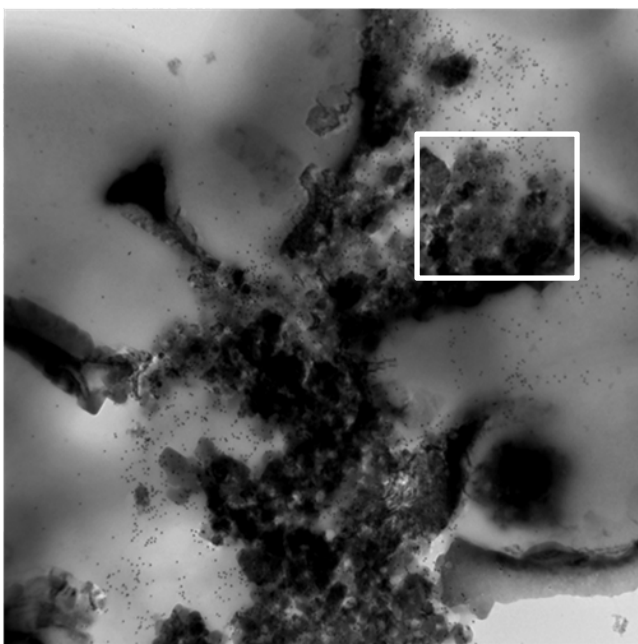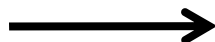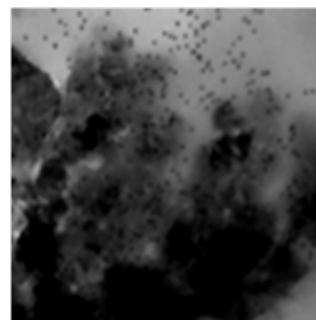

Supplement: Figure S3 — Higher magnification of immunogold particles on the surface of strain χ3339 flhCD (R995 + SPI-1 SipA-FLAG). Panel A: Image from Figure 6, Panel B in text. Panel B: Image from Figure 6, Panel E in text. (PDF) [file pone.0033080.s003.pdf]

**Figure S4.**

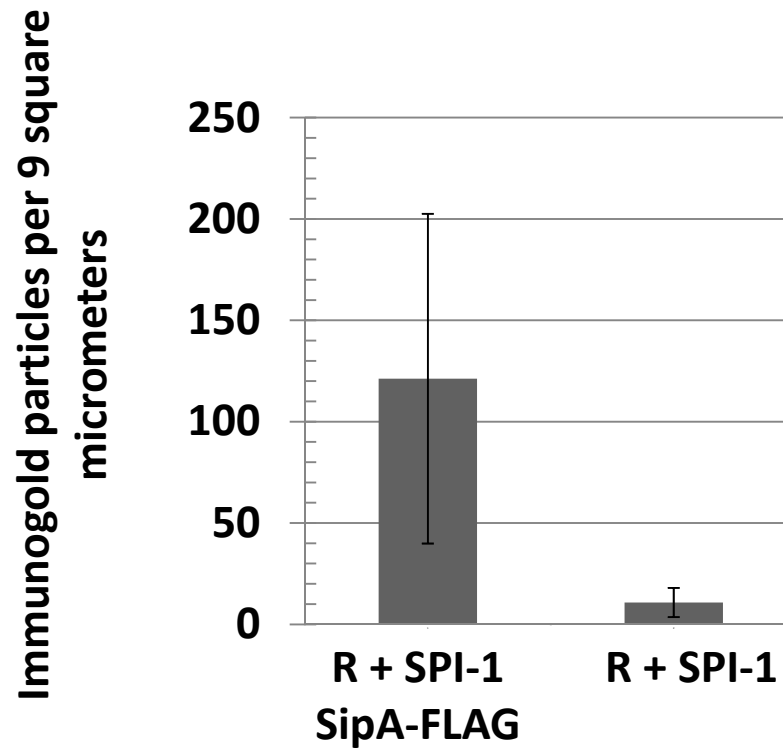

**p-value < 0.0001**

Supplement: Figure S4 — Quantification of immunogold particles observed per 9 square micrometers field of view via TEM of biofilm samples. Strains are χ3339 flhCD containing either R995 + SPI-1 or R995 + SPI-1 SipA-FLAG. Cultures were grown for biofilm formation, and then the biofilms were harvested and transferred to a grid for transmission electron microscopy as described in Materials and Methods. The number of immunogold particles per 9 square micrometers field of view were quantified. At least sixteen different fields of view were used per sample. (PDF) [file pone.0033080.s004.pdf]

Figure S5.

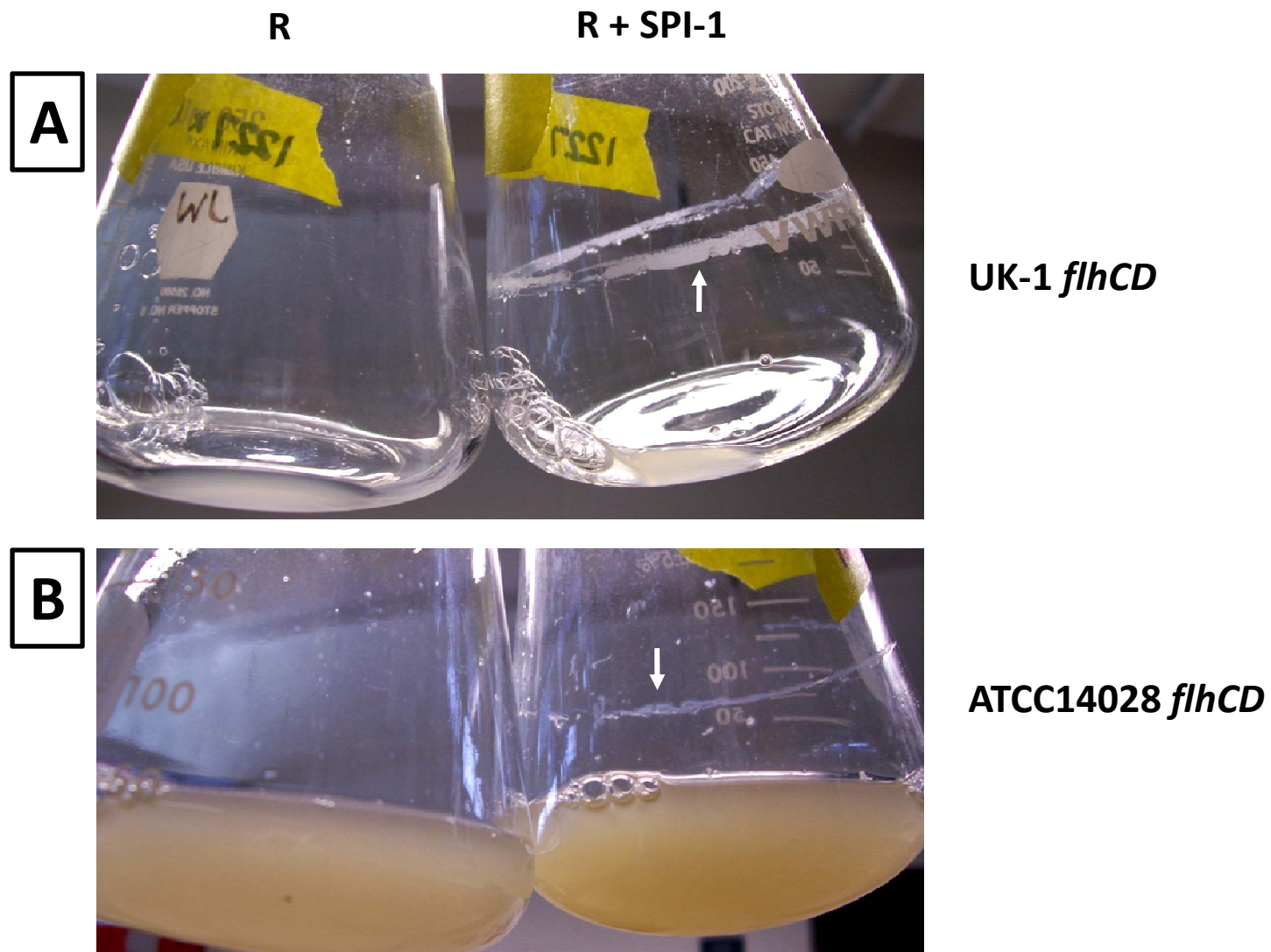

Supplement: Figure S5 — SPI-1 biofilms in S. Typhimurium UK-1 flhCD and ATCC14028 flhCD backgrounds. Panel A: Culture flasks of strain UK-1 flhCD containing either R995 or R995 + SPI-1. Panel B: Culture flasks of strain ATCC14028 flhCD containing either R995 or R995 + SPI-1. The white arrow indicates biofilm present in the R995 + SPI-1 cultures. (PDF) [file pone.0033080.s005.pdf]

Figure S6.

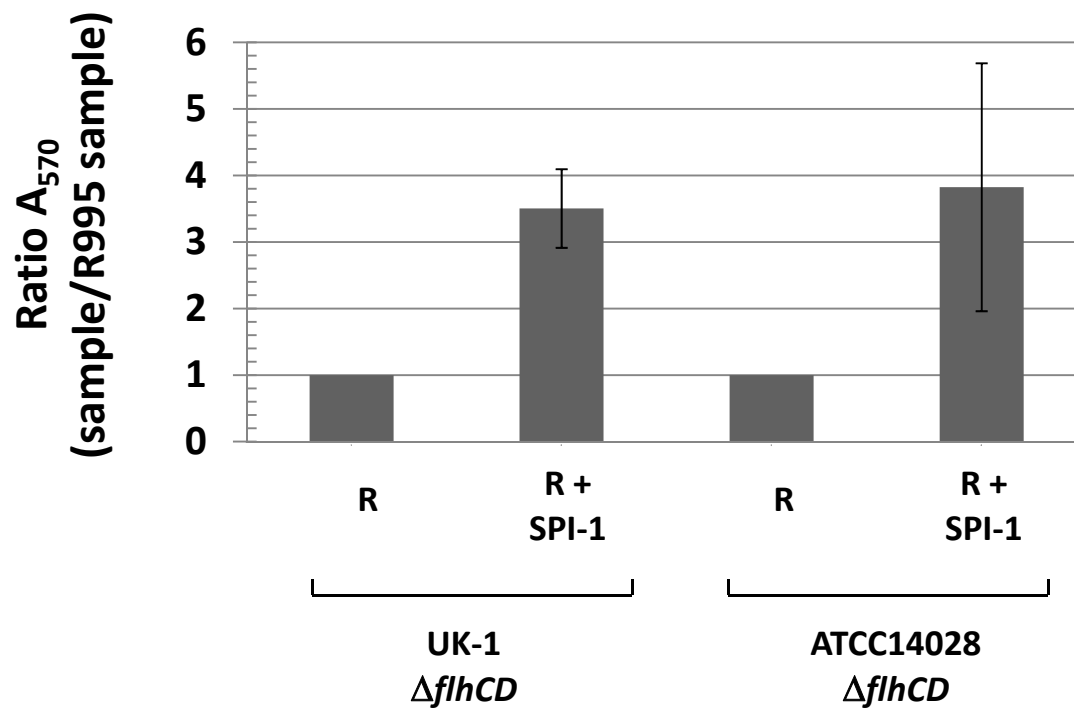

Supplement: Figure S6 — Quantification of crystal violet staining of SPI-1 biofilms in S. Typhimurium UK-1 flhCD and ATCC14028 flhCD backgrounds. Cultures as described in Figure S5 were processed for crystal violet staining as in the Materials and Methods. The A570 values were obtained from stained samples, and a ratio of A570 for each sample to the A570 for the R995 strain was calculated and plotted. The statistical difference between the R995 and R995 + SPI-1 samples for each strain was p = 0.0032 (UK-1 flhCD) and p = 0.0036 (ATCC14028 flhCD). At least four independent cultures were assayed for each strain. (PDF) [file pone.0033080.s006.pdf]

A

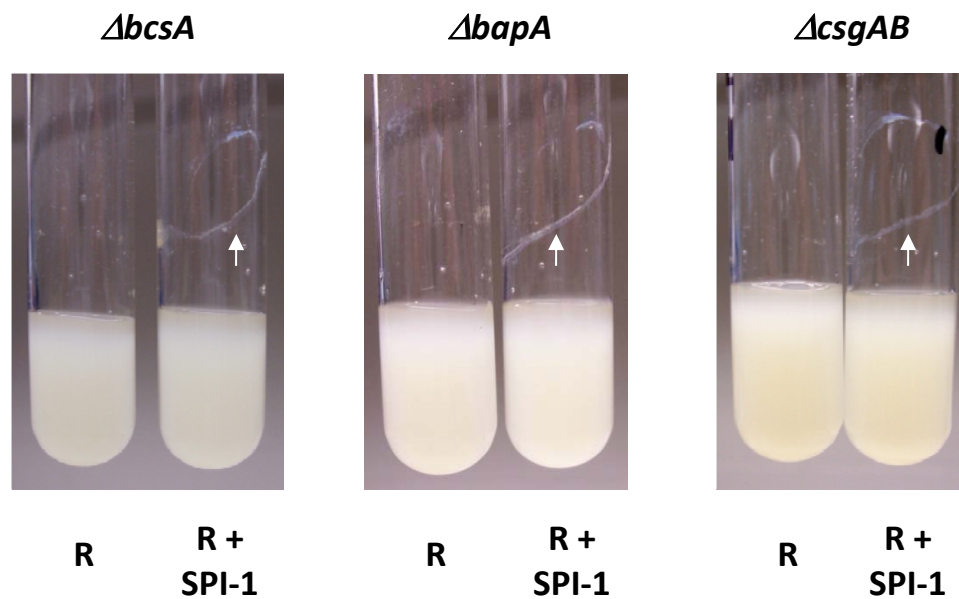

Figure S7.

B

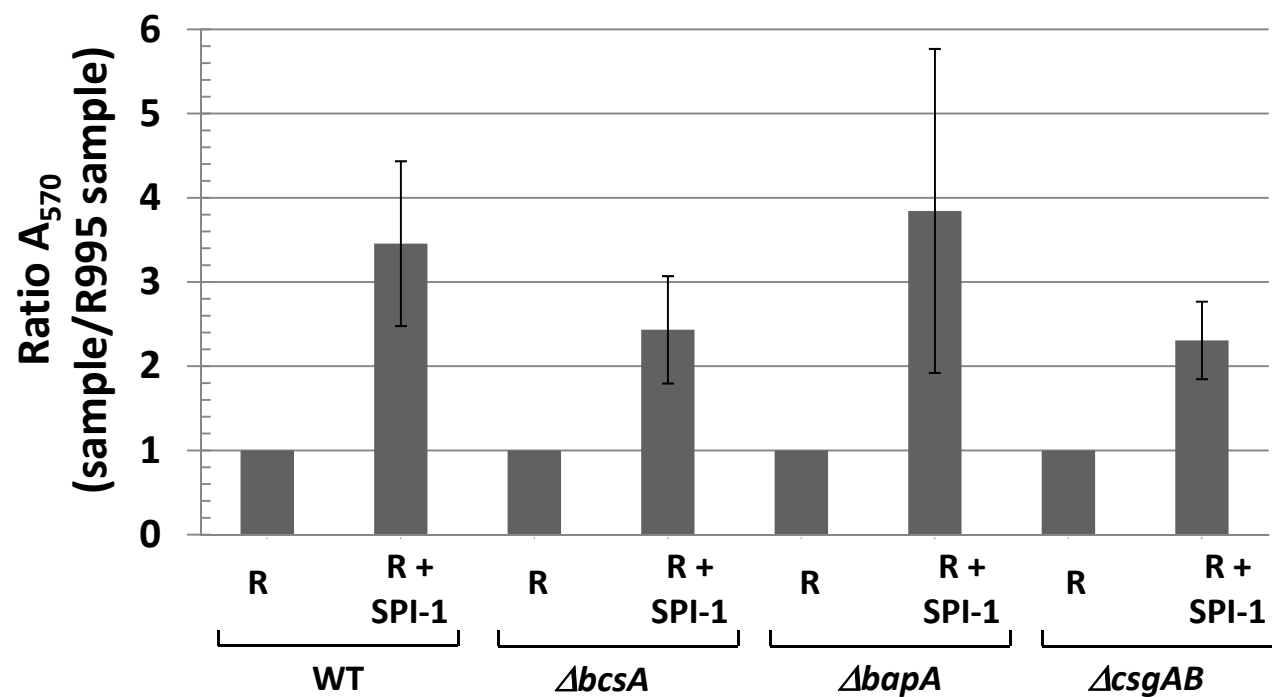

Supplement: Figure S7 — SPI-1 biofilms in S. Typhimurium χ3339 flhCD strains containing mutations in bcsA , bapA , and csgBA . Panel A. Cultures of the S. Typhimurium strain χ3339 flhCD containing mutations in bcsA, bapA, or csgBA and containing the plasmids R995 (denoted as “R”) or R995 + SPI-1 are shown. Note adhered biofilm for the R995 + SPI-1 strains indicated by white arrows. Panel B. Quantification of crystal violet staining. Cultures as described in panel A were processed for crystal violet staining as in the Materials and Methods. The A570 values were obtained from stained samples, and a ratio of A570 for each sample to the A570 for the R995 strain was calculated and plotted. The statistical difference between the R995 and R995 + SPI-1 samples for each mutant strain was p = 0.01 (bcsA), p = 0.0042 (bapA), and p = 0.004 (csgBA). At least four independent cultures were assayed for each strain. (PDF) [file pone.0033080.s007.pdf]

Figure S8.

A

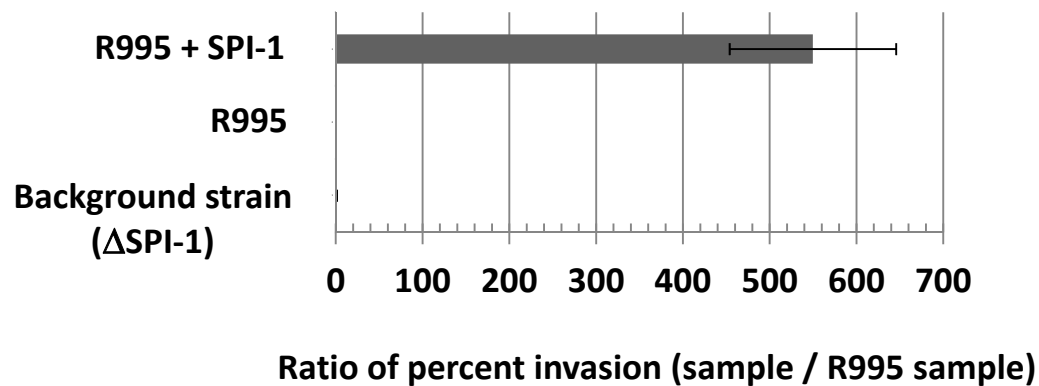

B

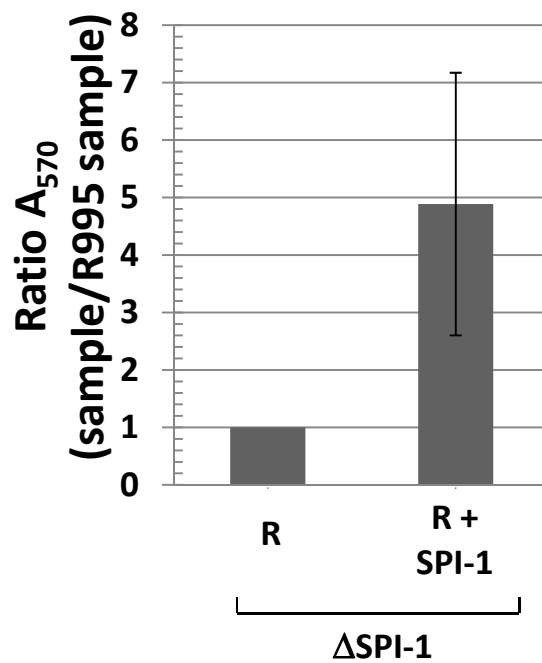

Supplement: Figure S8 — R995 + SPI-1 displays hyper-invasion and biofilm phenotypes in the absence of chromosomal SPI-1. Panel A: S. Typhimurium strain χ3339 flhCD ΔSPI-1 containing R995 + SPI-1 was tested for Int407 cell invasion compared to isogenic control strains containing either no plasmid or plasmid R995. The percent invasion of each strain (based on initial inoculum) was calculated. The data is presented as a ratio of the percent invasion for each strain to the percent invasion of the R995 strain. Note that the background strain is non-invasive due to the ΔSPI-1 mutation. Panel B: Cultures of strain χ3339 flhCD ΔSPI-1 containing either R995 or R995 + SPI-1 were processed for crystal violet biofilm staining as in the Materials and Methods. The A570 values were obtained from stained samples, and a ratio of A570 for each sample to the A570 for the R995 strain was calculated and plotted. The statistical difference between the R995 and R995 + SPI-1 samples was p = 0.001. Ten independent cultures were assayed for each strain. (PDF) [file pone.0033080.s008.pdf]
