# Supplementary material for: Characterization of Salmonella Type III Secretion Hyper-Activity Which Results in Biofilm-Like Cell Aggregation
Source: PLoS One. 2012 Mar 8;7(3):e33080. doi: 10.1371/journal.pone.0033080 (PMC3297627; doi:10.1371/journal.pone.0033080)
Supplement: Table S1 — Primers used to construct mutations using lambda Red recombination. (DOC) [file pone.0033080.s009.doc]

**Table S1. Primers used to construct mutations using lambda Red recombination.**

| **Primer name** | **Primer sequence** |
| --- | --- |
| p1flhcd5 | gccttcccggcgacatcacggggtgcggctacgtcgcacagtgtaggctggagctgcttc |
| 3flhcdp2 | gtccacaccgtttcggttaaacagcctgttcgatctgttccatatgaatatcctccttagttcc |
| 5bapap2 | atgcgtctactcgccgtggtttcgaaattgactggcgtctcatatgaatatcctccttagttcc |
| bapa3p1 | tgccggtgccgtcgcgatcgacggatatcaccgtattggctgtgtaggctggagctgcttc |
| 3bcsap1 | agccataacccgatccgacggctgtatcgccgcttgccgctgtgtaggctggagctgcttc |
| bcsa5p2 | atgagcgccctttcccggtggctgcttatcccgccggttacatatgaatatcctccttagttcc |
| 5csgbap2 | acaaattgttatttatgatgttgacaatactgggtgcgcccatatgaatatcctccttagttcc |
| csgba3p1 | aacctgacgcaccattacgctggaatcagatgcggtctgatgtgtaggctggagctgcttc |
